# Structure‐function relationships at the human spinal disc‐vertebra interface

**DOI:** 10.1002/jor.23627

**Published:** 2017-06-28

**Authors:** Britta Berg‐Johansen, Aaron J. Fields, Ellen C. Liebenberg, Alfred Li, Jeffrey C. Lotz

**Affiliations:** ^1^ University of California 513 Parnassus Avenue, S‐1157 San Francisco California 94143‐0514

**Keywords:** intervertebral disc, cartilage endplate junction, disc herniation, collagen, avulsion

## Abstract

Damage at the intervertebral disc‐vertebra interface associates with back pain and disc herniation. However, the structural and biomechanical properties of the disc‐vertebra interface remain underexplored. We sought to measure mechanical properties and failure mechanisms, quantify architectural features, and assess structure‐function relationships at this vulnerable location. Vertebra‐disc‐vertebra specimens from human cadaver thoracic spines were scanned with micro‐computed tomography (μCT), surface speckle‐coated, and loaded to failure in uniaxial tension. Digital image correlation (DIC) was used to calculate local surface strains. Failure surfaces were scanned using scanning electron microscopy (SEM), and adjacent sagittal slices were analyzed with histology and SEM. Seventy‐one percent of specimens failed initially at the cartilage endplate‐bone interface of the inner annulus region. Histology and SEM both indicated a lack of structural integration between the cartilage endplate (CEP) and bone. The interface failure strength was increased in samples with higher trabecular bone volume fraction in the vertebral endplates. Furthermore, failure strength decreased with degeneration, and in discs with thicker CEPs. Our findings indicate that poor structural connectivity between the CEP and vertebra may explain the structural weakness at this region, and provide insight into structural features that may contribute to risk for disc‐vertebra interface injury. The disc‐vertebra interface is the site of failure in the majority of herniation injuries. Here we show new structure‐function relationships at this interface that may motivate the development of diagnostics, prevention strategies, and treatments to improve the prognosis for many low back pain patients with disc‐vertebra interface injuries. © 2017 The Authors. *Journal of Orthopaedic Research*® Published by Wiley Periodicals, Inc. on behalf of Orthopaedic Research Society. J Orthop Res 36:192–201, 2018.

The intervertebral disc‐vertebra interface is structurally complex as it serves as the junction for multiple tissue types (Fig. 1A). Injury at this site has received growing interest due to its clinical significance. For instance, avulsion of the cartilage endplate (CEP) from bone at the cartilage‐vertebral endplate junction (EPJ) may be the most common form of disc injury since it is reportedly the initial site of failure in over 60% of intervertebral disc herniations (Fig. 1B).^1^, ^2^ Damage at the disc‐vertebra interface is highly innervated^3^ and is a specific imaging predictor of low back pain.^4^ The EPJ is intrinsically prone to failure because of stress concentrations that occur where material properties change abruptly.^5^, ^6^

**Figure 1:**
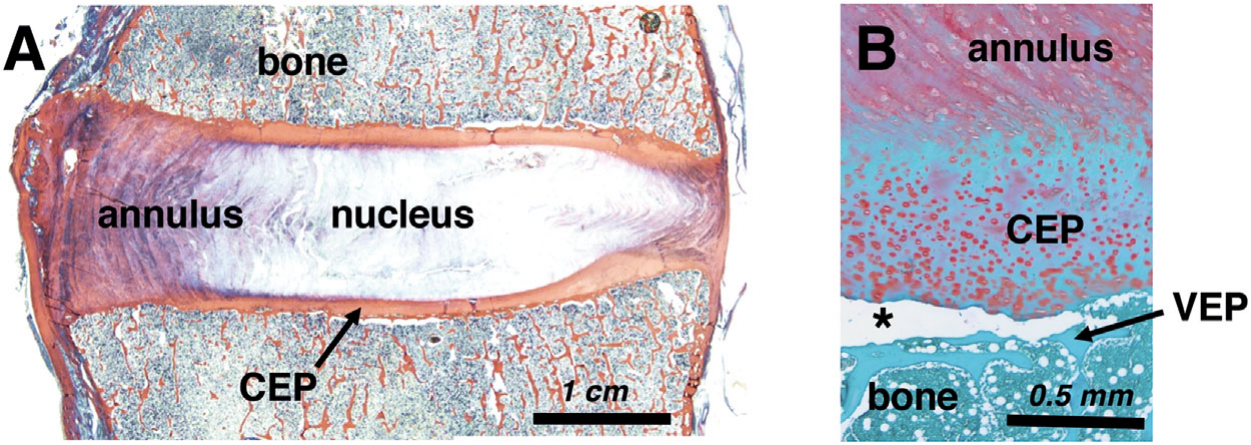
Histologic sections from human cadaveric lumbar spines. (A) Tri‐chrome Mallory–Heidenhain stained section depicting the annulus fibrosus, nucleus pulposus, vertebral bone, and cartilage endplate (CEP). (B) Safranin‐O stained section illustrating an in situ CEP avulsion (at asterisk) from the underlying vertebral endplate (VEP).

Despite its clinical significance, the biomechanical behavior and structural risk factors of the disc‐vertebra interface remain underexplored. Ex vivo imaging studies have shown that collagen fibers of the outer annulus extend into adjacent vertebrae and serve to anchor the disc to the bone at the vertebral rim.^7^, ^8^ By contrast, fibers of the inner annulus and nucleus integrate with the CEP and sometimes extend into the calcified cartilage layer,^7^, ^9^, ^10^ but the CEP shows little to no integration with the underlying bone. Historical data suggest structural weakness at the cartilage‐vertebral EPJ, as supported by recent work showing this location as the weak link of the disc‐vertebra interface.^11^

The architectural features and biomechanical behavior of other hard‐soft tissue interfaces in the body inform hypotheses about structural properties of the disc‐vertebra attachment. For example, functional grading of material properties (i.e., mineralization and collagen fiber organization) enables effective stress transfer and decreases stress concentrations across tendon‐bone interfaces,^5^, ^6^, ^12^ while collagen fiber anchoring of articular cartilage to bone enhances the integrity of attachment.^13^ It remains unclear whether similar mechanisms are in place to enhance disc‐vertebra integrity.

The goal of the current study was to perform an in‐depth analysis of the failure mechanisms of the human disc‐vertebra interface and the associated structural features related to collagen fiber anchoring and functional grading. Specifically, we sought to: (i) identify failure mechanisms at the disc‐vertebra interface and measure the failure strength and local strain profiles of human bone‐disc‐bone specimens using biomechanical tension tests; (ii) assess the degree of collagen fiber anchoring and functional grading across the EPJ using polarized light microscopy, SEM, and μCT; and (iii) perform structure‐function correlations to assess which structural features contribute to interface strength. Insight into mechanisms of endplate integrity and features associated with weakness could help inform new diagnostic tools and more effective treatments for patients with pain due to pathologic EPJs.

## MATERIALS AND METHODS

### Sample Preparation

**Figure 2:**
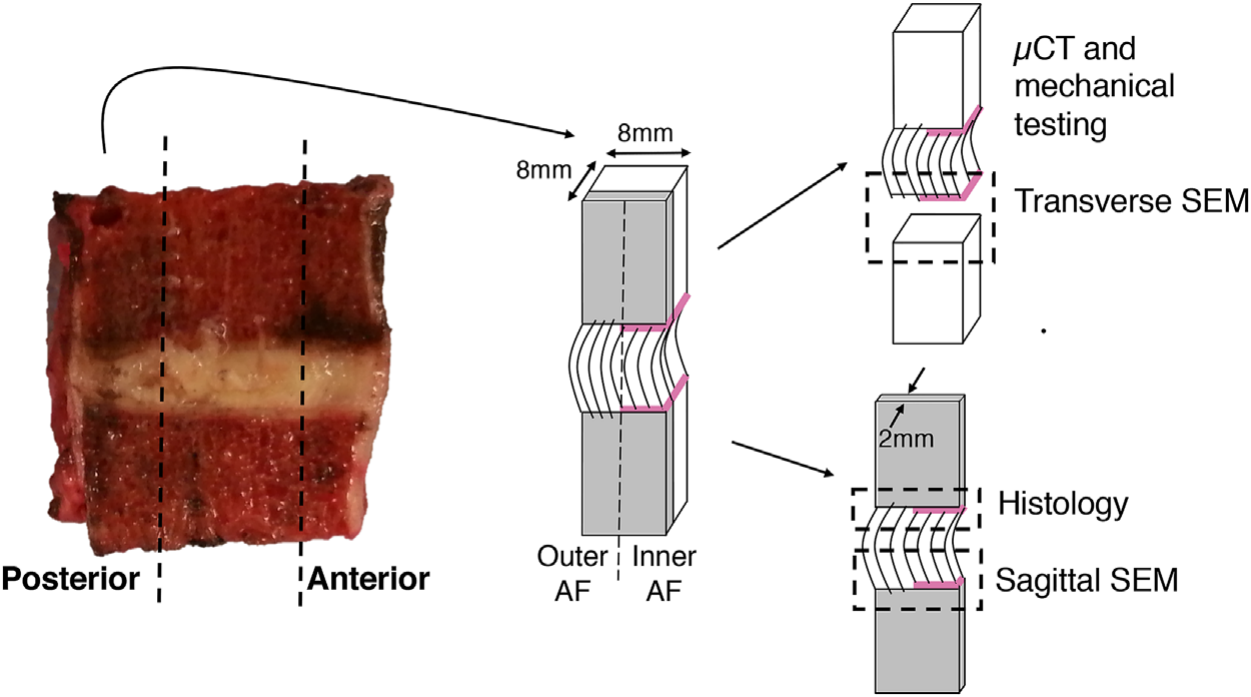
Sample preparation and allocation. Bone‐disc‐bone specimens were cut from the posterior and anterior annulus regions to include both outer and inner annulus fibrosus (AF). A thin sagittal slice was cut for sagittal‐plane histology and SEM, while the bulk sample was scanned with μCT and mechanically tested. Failure surfaces were scanned with SEM. Note that annulus fibers of the outer AF attach directly to the vertebra, while those in the inner AF attach to the cartilage endplate (denoted by pink line).

Seventeen cadaveric motion segments were isolated from seven human spines (levels T10‐L1, 4M/3F, 49–65 years old) within 72 h post‐mortem (UCSF Willed Body Program). Each motion segment was sectioned on a band saw into ∼8 mm‐thick parasagittal slabs, which were visually classified by two raters using the Thompson grading scheme (Grade 1 = healthy; Grade 5 = severely degenerated),^14^ with good inter‐rater reliability (kappa = 0.69). Images from one spine (three motion segments) were unavailable for Thompson grading. From the central‐most parasagittal slab of each motion segment, cuboidal bone‐disc‐bone specimens including both outer and inner annulus (∼8 × 8 mm^2^ in cross‐section) were extracted from the posterior annulus (PA) and anterior annulus (AA) regions. Specimens were held in place with wooden blocks while cutting. No visible tears were caused by the sectioning process. Tissue was kept hydrated by spraying with phosphate‐buffered saline (PBS) throughout preparation. Six specimens showed existing fissures in the annulus or separations at the EPJ, and were not used for subsequent analyses. One 2 mm‐thick parasagittal slice from each sample was fixed in 10% formalin for histology and scanning electron microscopy (SEM). The remainder of each specimen was designated for μCT and mechanical testing (Fig. 2).

### Micro‐Computed Tomography (μct)

Prior to mechanical testing, specimens were thawed gradually on ice and scanned in PBS at a 15 μm voxel size using a tube voltage of 55 kVp and an X‐ray intensity of 109 μA (μCT 50, SCANCO Medical, Brüttisellen, Switzerland). The average duration of the scans was 90 min. Image reconstruction was performed using SCANCO software.

A lower threshold of 300 grayscale units was used to segregate mineralized tissue from soft tissue and scan medium, and Gaussian filters with a sigma of 0.5 and support of two were applied to remove noise. Morphometric indices were calculated using a 3D model‐independent algorithm, and tissue mineral density (TMD) was calculated using a linear attenuation coefficient determined by calibration with a SCANCO hydroxyapatite phantom. SCANCO Evaluation Program v6.5–3 was used to quantify bone volume fraction (BV/TV), TMD, and trabecular microarchitecture (trabecular number, thickness, and spacing) on a 3 × 3 × 3 mm^3^ central cube of trabecular bone 3 mm from the EPJ that failed in mechanical tests (superior or inferior).

**Figure 3:**
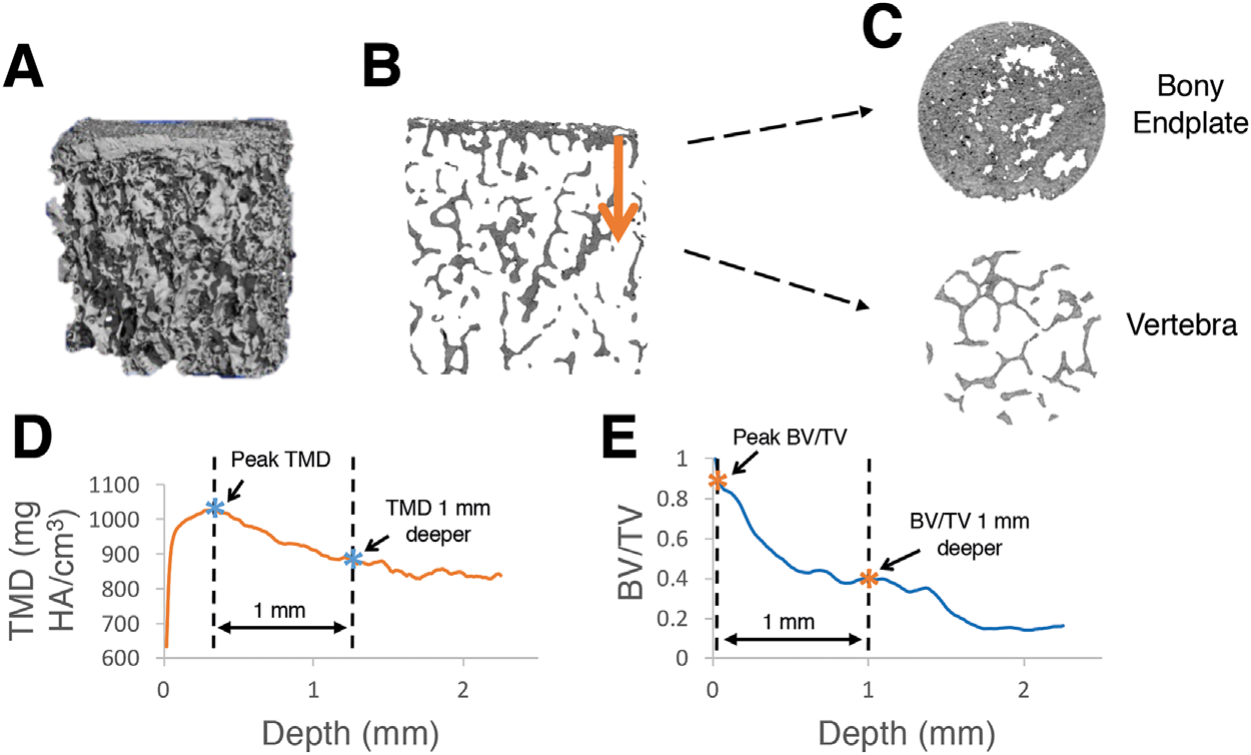
Process of calculating gradients in tissue mineral density (TMD) and trabecular bone volume fraction (BV/TV) at the endplate junction regions. (A) Three‐dimensional μCT data is loaded into custom IDL algorithm, (B and C) algorithm traverses from the dense bony endplate into the deeper vertebral bone, and average values of (D) TMD, and (E) BV/TV are plotted by depth. Differences between peak values (first asterisk) and values 1 mm deeper (second asterisk) were used to assess local gradients in TMD and BV/TV. Note that (B) and (C) are slices in the sagittal and transverse planes, respectively.

Next, a custom script was written in Interactive Data Language (IDL) to quantify TMD and BV/TV at the inferior and superior vertebral endplate regions for each specimen. The algorithm performs the following steps: (i) reads in TMD data; (ii) thresholds and masks the bone; (iii) crops the central 2.6 mm‐diameter core of the sample; (iv) marches in the axial (Z) direction—beginning above the endplate—and identifies the endplate surface by finding the first non‐zero pixel for each X‐Y position; and (v) stores TMD values at every pixel for the next 2.25 mm depth. Then, the average TMD and BV/TV across the specimen were calculated and saved at every 0.015 mm from the endplate to the 2.5 mm depth. A custom MATLAB script was then written to calculate endplate TMD and BV/TV (defined as the average TMD and BV/TV in the first 0.2 mm‐thick region from the surface) and gradients in TMD and BV/TV (defined as the difference between (i) the peak TMD and BV/TV, and (ii) the TMD and BV/TV 1 mm deeper). The 1 mm distance spans the thickness of the vertebral endplate, which is typically between 0.4 and 1.0 mm thick.^15^, ^16^, ^17^ This process is outlined visually in Figure 3.

### Mechanical Testing

**Figure 4:**
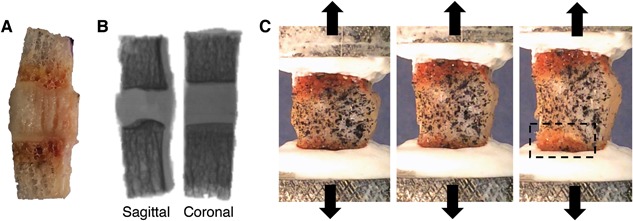
Specimen is (A) cleaned of bone marrow, (B) X‐rayed in two orthogonal planes to calculate geometry, and (C) speckle‐coated on parasagittal surface, mounted in custom‐designed aluminum pots, and pulled in tension to failure. Box in (C) shows location of endplate junction failure. A, B (left), and C are all sagittal views with the inner annulus on the left and outer annulus on the right.

**Figure 5:**
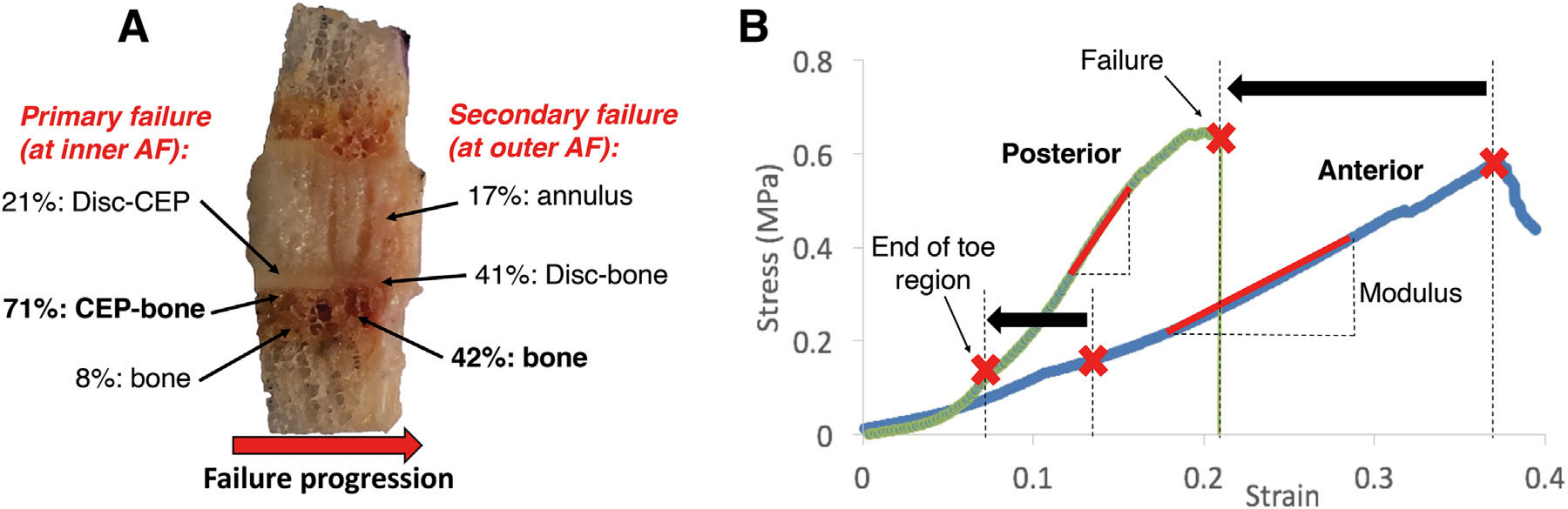
(A) Distribution of failure locations at inner annulus fibrosus (AF) and outer AF regions, with red arrow showing typical direction of failure progression from inner to outer AF. CEP, cartilage endplate; (B) Stress‐strain curves for representative posterior annulus and anterior annulus specimens. Posterior specimens had a shorter toe region and lower failure strain (denoted by black arrows) than anterior specimens, as well as a higher modulus than anterior specimens (*p *< 0.01). Strains on x‐axis are grip‐to‐grip strains along the loading axis.

Specimens were cleaned of marrow using a water jet, X‐rayed in two orthogonal planes to measure cross‐sectional area, speckle‐coated on one parasagittal surface with Verhoeff's tissue stain for DIC, and affixed to a custom testing fixture using casting resin (Fig. 4). Specimens were kept hydrated by spraying with PBS throughout preparation. Specimens were preconditioned for five cycles between 0 and 5% tensile strain, then loaded to failure in tension at 0.01 mm/s on a mechanical load frame (ElectroForce 3200; Bose, Eden Prairie, MN). The side of the disc where each specimen failed (inferior or superior) was recorded. Force‐displacement data were normalized into stress‐strain data using cross‐sectional area (measured on X‐rays as an average of cross‐sectional area at the inferior endplate and superior endplate) and initial specimen height (measured with calipers as the average length between the two endplates along the center axis of the specimen's four faces). Measured cross‐sectional areas and initial specimen heights were used to normalize the force‐displacement data into stress‐strain data. A MATLAB algorithm was written to calculate (i) toe region strain, defined as the strain where the stress‐strain curve transitions to the linear portion (*R* ^2 ^> 0.99); (ii) tensile modulus, defined as the slope of the stress‐strain curve during the linear portion; and (iii and iv) failure strength and strain (Fig. 5). The toe region has been previously reported,^18^, ^19^ and results from viscoelastic straightening of initially curved annulus collagen fibers. Mechanical tests were unreliable for seven specimens (i.e., casting resin slipped and/or machine became unstable) and thus the data for these specimens were not used for subsequent analyses.

Failure locations were observed visually during testing (Fig. 4C). Videos captured during tension tests at 30 frames/s were watched in slow‐motion to confirm initial failure location and observe failure progression. Failed specimen surfaces were visualized after testing to confirm precise failure locations (as determined by tissue types on either side of the failure).

### Surface Strain Tracking

**Figure 6:**
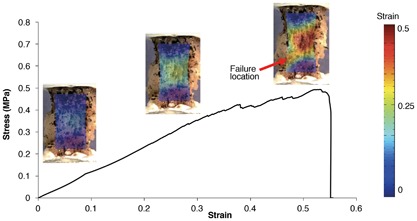
Stress‐strain curve for a bone‐disc‐bone specimen. Strains on x‐axis are grip‐to‐grip strains along the loading axis. Principal strain maps are overlaid on specimen images acquired at corresponding levels of applied strain.

Videos of tension tests (1920 × 1080 pixels, 30 frames/s) were analyzed using DIC. A grid mesh was created on the front‐facing speckle‐coated sagittal tissue surface. This mesh and sequential images of surface deformation (sampled at one frame per 5 s) were converted to principal strain profile maps using strain tracking code provided by the Victor Barocas Group at the University of Minnesota.^20^ Speckle‐coating and tracking parameters were optimized such that tracked subsets were ∼15 × 15 pixels and individual speckles were ∼3–5 pixels in diameter. This ratio of speckle size to subset size has been shown to produce high accuracy for DIC^21^ and allowed us to track local strains across the CEP, which was typically ∼1 mm thick (∼30–40 pixels). The error in strain measurements is a function of the optical resolution, speckle size, and subset size, which for our particular settings was in the range of 10–100 μm. Strain maps were overlaid on specimen test videos (Fig. 6). A MATLAB algorithm was written to plot principal strain at each central axial location across the specimen. Principal strains were analyzed—rather than axial strains—due to the nonhomogeneous nature of the tissue samples. Strain gradients were calculated as the difference between the maximum strain (in the annulus) and minimum strain (in the bone), as a surrogate measure of functional grading across the interface.

### Scanning Electron Microscopy (SEM)

Following fixation, the adjacent 2 mm‐thick parasagittal slices (untested) were separated into superior and inferior halves. The half corresponding to the side that failed mechanically was evaluated with SEM. These untested parasagittal sections, along with transverse failure surfaces of the samples tested mechanically, were dehydrated in an ethanol series with ascending concentrations. Scans of parasagittal sections and transverse failure surfaces were acquired using a Zeiss Sigma 500 scanning electron microscope (Carl Zeiss International, Thornwood, NY).

### Histology

The remaining half of each untested parasagittal slice was decalcified in EDTA, dehydrated in ethanol, embedded in paraffin, and cut into 7‐μm thick sections. Three to five sections from each specimen were stained with a tri‐chrome Mallory–Heidenhain stain containing aniline blue, orange G, and acid fuchsin to observe gross morphology and to measure CEP thickness. CEP thickness was measured at the inner‐most edge of each specimen using ImageJ. The inner‐most edge was chosen because this was the location of initial mechanical failures. Three to five additional sections from each specimen were stained with Picrosirius Red to visualize collagen fiber architecture at the EPJ using polarized light microscopy.

### Statistics

Statistical analyses were performed using JMP (SAS Institute, Inc., Cary, NC). Paired two‐tailed *t*‐tests were used to assess (i) differences in mechanical properties and structural properties between posterior and anterior regions; and (ii) differences in structural properties between failed and intact sides of each specimen. Univariate linear regression analysis was performed to assess correlations between mechanical properties and independent variables (structural properties, disc grade/height, and age/sex). Multiple linear regression was used to assess relationships between each mechanical property (toe region strain, modulus, failure strength, and failure strain) and independent structural variables. Least‐squares estimation with backward elimination was used to predict each mechanical property, where only variables with significant slopes were retained in the model. Significance for all statistical tests was defined by *p *< 0.05.

## RESULTS

### Disc Height and Thompson Grade

The average whole disc height and isolated specimen height were 7.1 ± 1.5 mm and 6.9 ± 1.2 mm, respectively. All discs were Thompson grades 2, 3, and 4 (average 2.6).

### Micro‐Computed Tomography

Bone properties measured from central trabecular cubes demonstrated that posterior specimens had a 13% higher tissue mineral density (*p *< 0.005) and 21% lower trabecular spacing (*p *< 0.005) than anterior specimens. This agrees with previous studies showing that bone is denser in the posterior regions.^22^, ^23^ Local analyses at the endplate regions revealed a gradient of decreasing BV/TV and TMD from the vertebral endplate into the trabecular bone (Fig. 3). Average BV/TV and TMD in the vertebral endplates were 0.72 ± 0.10 and 782.9 ± 67.8 mg HA/cm^3^, respectively. BV/TV gradients and TMD gradients (between the location of peak value and 1 mm deeper) were 0.82 ± 0.07 and 53.4 ± 41.6, respectively. BV/TV in the endplates was significantly correlated with BV/TV in the central trabecular bone (*R* ^2^ = 0.23, *p* = 0.03).

### Mechanical Testing

Mechanical failures initiated near the disc‐vertebra interface for all specimens (52% at the inferior side and 48% at the superior side). Failure initiated at the inner annulus region and progressed toward the outer annulus region. A total of 71% of primary failures (at the inner annulus region) occurred at the CEP‐bone interface, with CEP being stripped from the underlying bone. A total of 21% occurred at the disc‐CEP interface, and 8% occurred within the subchondral bone. Following primary failure at the inner annulus region, failure progressed to the outer annulus region, where 42% of secondary failures occurred mid‐bone, 41% at the disc‐bone interface (note: no CEP is present between the disc and bone in this out annulus region), and 17% within the annulus (Fig. 5). The average modulus, strength, and failure strain were 2.08 ± 1.49 MPa, 0.43 ± 0.16 MPa, and 38.5 ± 20.3%, respectively.

Posterior specimens exhibited a 58% shorter toe region (4.5 ± 1.5% vs. 10.6 ± 6.2%, *p *< 0.01), 50% higher modulus (3.0 ± 1.7 MPa vs. 1.2 ± 0.5 MPa, *p *< 0.005), and 58% lower failure strain (21.3 ± 10.3% vs. 51.1 ± 16.0%, *p *< 0.0005) than anterior specimens (Fig. 5). Failure strength was similar between anterior and posterior regions (*p *> 0.25).

Across all specimens, the highest principal strains occurred mid‐annulus, while the lowest occurred in the bone. The strain gradient (measured as the difference between the maximum and minimum principal strains) became steeper with higher applied strains. At very high applied strains (near failure), high local strains were observed at the failure location (Fig. 6).

### Scanning Electron Microscopy

**Figure 7:**
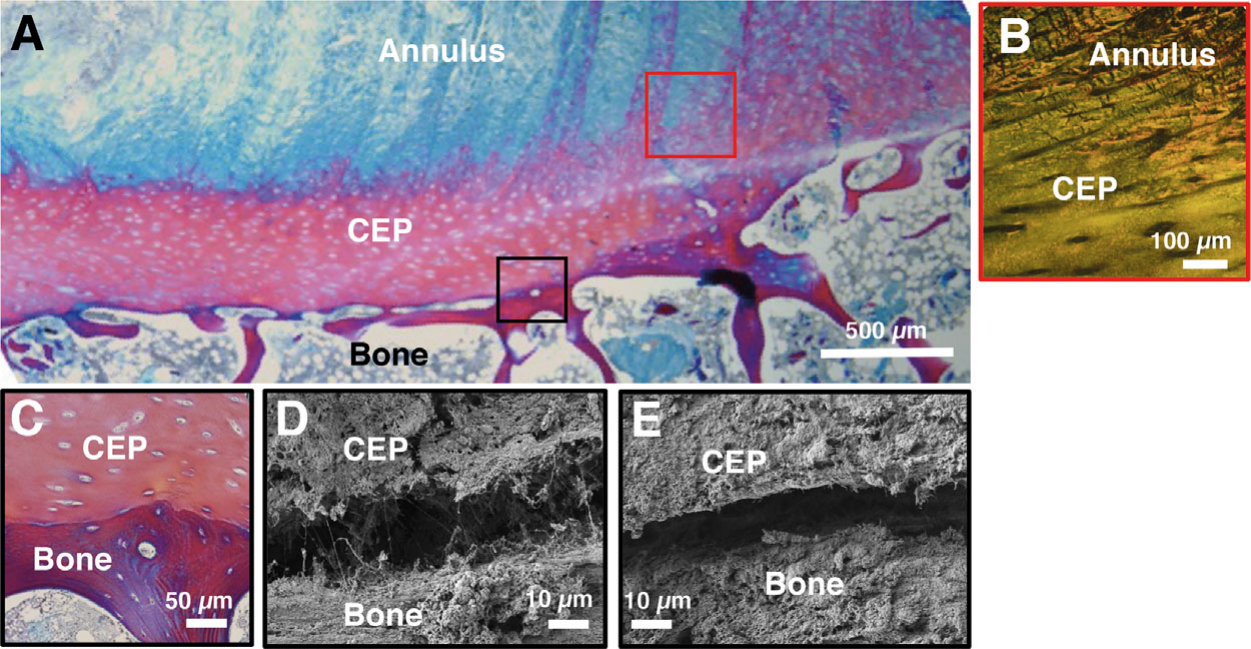
Sagittal histology (A,B,C) and SEM (D,E) images of the endplate junction. (A,C) are stained with trichrome stain, and (B) is stained with picrosirius red and visualized under polarized light. (B) shows annulus fibers integrating with the cartilage endplate (CEP), while (C) shows a clear demarcation between CEP and bone with no integration. (D and E) show small gap between CEP and bone using SEM. Black and red boxes on (A) are color‐coded to correspond with approximate regions of (B–E).

**Figure 8:**
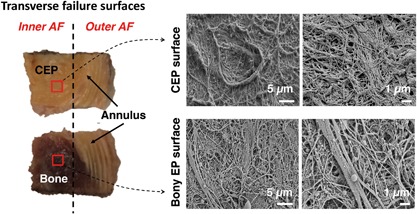
(Left) Transverse failure surfaces for specimen that failed at cartilage endplate (CEP)‐bone interface at inner annulus fibrosus (AF) region and failed in mid‐annulus at outer AF region. Red squares indicate location of SEM scans; (Right) SEM scans of failure surfaces imaged for CEP; and opposing bony endplate surface.

SEM scans in the sagittal plane indicated a lack of structural integration between the CEP and bone. Many specimens showed a physical gap (5–30 μm thick) between the CEP and bone (Fig. 7D and E). The gap typically either had sporadic thin collagen fibrils showing evidence of a weak connection (Fig. 7D) or was void of collagen fibers altogether (Fig. 7E). Transverse failure surfaces showed a bed of collagen fibers oriented in the plane of the failure interface (Fig. 8). For specimens that appeared to fail at the CEP‐bone interface, the vertebral endplate surface displayed cartilage‐like fiber morphology, indicating that the specimen failed between the CEP, and the calcified cartilage layer.

### Histology

Histologic sections also indicated a lack of structural integration between the CEP and adjacent bone (Fig. 7). Inner annulus fibers joined at oblique angles with the CEP (Fig. 7A and B), but did not anchor into the subchondral bone (Fig. 7C). The outer annulus region did not contain CEP, and fibers anchored directly into the vertebral rim as previously described. The average CEP thickness was 0.85 ± 0.21 mm, as measured at the inner edge of each specimen in ImageJ.

### Structure‐Function Relationships

**Figure 9:**
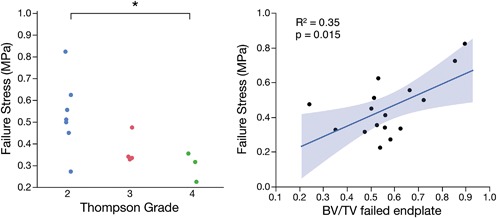
(Left) Failure stress was significantly lower in samples from discs with Thompson Grades 4 versus 2 (*p = 0.026, *t*‐test); (Right) Failure stress was positively correlated with trabecular bone volume fraction (BV/TV) in the vertebral endplate that failed during mechanical tests (*R* ^2^ = 0.35, *p* = 0.015).

Various relationships were found between structural and mechanical properties. Failure stress was significantly lower in specimens with a Thompson Grade of 4 than those with a Thompson Grade of 2 (*p* = 0.026, *t*‐test; Fig. 9). Interestingly, failure stress significantly increased with BV/TV in the failed endplate (*R* ^2^ = 0.35, *p* = 0.015; Fig. 9). Within each specimen, the endplate (inferior or superior) that remained intact during mechanical tests had 8% higher BV/TV and 3% higher TMD than for the endplate that had failed (*p* < 0.05). The intact side also tended to have a 5% lower (less steep) BV/TV gradient (*p* = 0.08).

Failure strength tended to increase with decreasing CEP thickness, but this relationship was not significant (*R* ^2^ = 0.22, *p* = 0.17). However, Thompson Grade and CEP thickness were both significant predictors of failure stress in a multiple linear regression model (*p* = 0.03 for both). This multiple linear regression model was statistically significant, with 68% of the variation in failure stress explained by the predictors (overall *p* = 0.02). Neither failure strength nor modulus were significantly associated with age, sex, or strain gradients across the endplate (measured by surface strain tracking). However, toe region strain decreased with age (*p* = 0.03), and both toe region strain and failure strain increased with steeper strain gradients (*p* = 0.08 and *p* = 0.02, respectively). No additional significant relationships were found between structural properties and toe region strain, modulus, or failure strain.

## DISCUSSION

**Figure 10:**
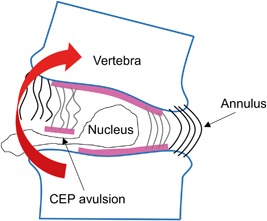
One proposed mechanism of disc herniation, in which cartilage endplate (CEP) is avulsed from bone, allowing disc material to escape. Endplate junction failure is the most common cause of clinical disc herniations^1^ and may occur during spinal movements involving bending motions that place the CEP‐bone interface in tension.

Our findings indicate that the CEP and vertebra at the inner annulus region are not well‐integrated, which is supportive of previous findings^24^, ^25^, ^26^ and may explain the weakness of this zone. Because the CEP and inner annulus are structurally integrated, tension at the CEP‐bone interface generated during certain spinal movements can avulse the cartilage from the underlying bone (Fig. 10).

Experimentally, we observed that 71% of primary failures occurred at the CEP‐bone interface at the inner annulus region, which is consistent with reports that 65% of clinical disc herniations initiate at the CEP‐bone interface.^1^ After initial failure, the failure surface propagated to the outer annulus region, where secondary failures occurred mostly within the annulus or bone. This shift from an interface failure to a tissue substance failure may be attributed to the firm anchoring of the outer annulus fibers into the underlying bone at the vertebral rim,^7^, ^8^ which creates a strong connection and transfers the vulnerability away from the interface. This is further supported by whole motion segment bending tests showing that the peripheral annulus fibers remains attached to the vertebral rim, while the middle annulus commonly pulls cartilage away from the underlying bone.^27^ This may mimic failure progression in vivo during herniation.

We have reported new structure‐function relationships at the disc‐vertebra interface. We observed that bone density of the vertebral endplates plays an important role in the strength of the disc‐vertebra interface, as evidenced by: (i) a positive linear correlation between BV/TV and failure strength; and (ii) significantly higher BV/TV and TMD in the intact (unfailed) end versus the failed end of each specimen. These findings suggest that denser bone in the vertebral endplate creates a larger surface area of attachment to the CEP, thereby strengthening the interface.

Importantly, we also found significant relationships between clinically measurable parameters and failure strength. Peripheral CEP thickness can be measured clinically with new imaging techniques that can visualize the CEP, such as fast low‐angle shot (FLASH) MRI^28^ and ultrashort echo‐time (UTE) MRI.^29^, ^30^, ^31^ Our findings suggest that disc degeneration grade and CEP thickness may serve as potential clinical risk measures for avulsion‐type herniation. However, the sample size was limited, and future studies should further assess these correlations in human subjects. We did not find a significant effect of age on failure strength, which may be related to the narrow age range in this study or the fact that disc degeneration occurs at different rates. Interestingly, CEP thickness was a negative predictor of failure strength. This may be due to a thicker CEP creating a larger separation between the annulus and bone, thereby reducing structural integration between the annulus and bone. Lastly, we found that BV/TV in the failed endplate (which was a significant predictor of failure strength) was significantly correlated with trabecular BV/TV in the vertebra, indicating that clinical quantitative computed tomography (QCT) or dual‐energy X‐ray absorptiometry (DXA) measures of vertebral bone quantity may also be predictive of disc‐vertebra injury risk.

Although the disc‐vertebra interface bears complex loads including shear, compression, tension, and torsion, we applied uniaxial tension in order to examine the cohesive properties of the EPJ. Further, tension at this interface may be particularly important since it is known that forward bending is the main contributor to disc herniation injuries, which places the PA into tension.^27^, ^32^ Moreover, sheep discs subjected to axial tension contain more EPJ failures than those subjected to torsion or in‐plane tension.^33^ Failure stresses and failure locations measured in the current study were consistent with those found in previous human disc‐bone tensile tests. Our measured failure stresses (range: 0.22–0.93 MPa) were within the range of those reported by Balkovec et al.^11^ (range: 0.14–2.8 MPa) and lower than those found by Green et al.^18^ (average 1.7 MPa for posterior and 3.8 MPa for AA), presumably because we cut our specimens smaller to isolate more specific regions, thus disrupting the collagen network mid‐fiber due to the angled orientation of annulus fibers. The higher modulus of the PA compared to the AA may help to resist forward bending movements, but the lower extensibility may put weak EPJs at risk for herniation.

Microstructural imaging showed very little structural integration between the disc and vertebra. SEM showed 5–30 μm discernible gaps between the CEP and bone with no ligament‐like bridging other than thin individual collagen fibrils (Fig. 7D and E). These gaps may be a result of interlaminar delamination caused by scanning under vacuum (low pressure), which is a known artifact of electron microscopy and has been observed in previous studies.^10^, ^34^ Delamination was not observed in any other regions of the specimens, and provides additional evidence of the fragility of this interface.

The weakness of the CEP‐vertebra interface is in stark contrast to other cartilage‐bone interfaces, such as the articular cartilage‐bone interface and ligament and tendon insertions, where collagen fibers are oriented perpendicular to the interface and anchor the soft tissue to the bone.^12^, ^13^, ^35^ Collagen fiber anchoring at the CEP‐vertebra interface may be unnecessary in healthy discs with normal loading, as high nucleus pressure keeps the cartilage pressed onto the bone and prevents tension from developing across the interface.^2^, ^36^ However, when a disc is subjected to very high bending loads, tensile forces in the inner annulus may cause the CEP to avulse. Degenerated discs with diminished nuclear pressure may be at a higher risk of CEP avulsion. This is supported by studies showing that CEP fragments found in disc herniations were associated with increasing age and degeneration.^37^, ^38^, ^39^ Therefore, although many disc herniations occur in younger individuals with non‐degenerated discs,^40^ avulsion‐type herniations may occur more commonly in the elderly.

**Figure 11:**
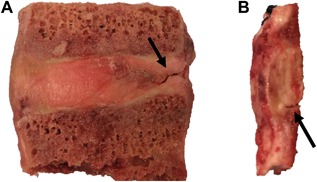
Un‐tested lumbar specimens. Specimens are often too degenerated for mechanical testing and show (A) annular fissures and (B) separation at the CEP‐bone interface.

A significant limitation of this study is that we tested only thoracic levels because most lumbar levels of the donor spines contained annular fissures and existing defects at the CEP‐bone interface that precluded harvesting intact specimens for analysis (Fig. 11). The majority of clinical disc herniations occur in the lumbar spine.^41^ Although we tested only lower thoracic levels (T10–L1), we expect that our conclusions regarding the importance of structural integration applies to lumbar specimens, which have similar endplate morphology. Moreover, the annular fissures and defects at the CEP‐bone interface in the lumbar levels further evidences the weakness of this interface in vivo.

Another limitation of this study is that deformation fields were only measured on the front‐facing parasagittal surface during tension testing. Because of this, initial failures that occurred mid‐section or at the opposite parasagittal surface were not captured in videos. This is an inherent limitation of 2D strain tracking on 3D tissue. However, we were able to determine when initial failure occurred from drops in force‐displacement curves and correlate failure strength to our measured structural properties. Furthermore, the general location of initial failure (i.e., inner vs. outer annulus) was evident by visually observing failure progression as samples were pulled to complete failure, and precise locations of failure were evident by observing the failed surfaces following testing.

Functional grading of material properties at interfaces of dissimilar biological materials is a mechanism of smoothing structural discontinuities and decreasing stress concentrations. Examples of this are the grading of mineral and collagen at the tendon‐bone interface,^5^, ^6^, ^12^, ^42^ grading of structural and chemical properties of the bone‐tooth interface,^43^ and grading of fiber orientation in the tissue encasing blood vessels in bone.^44^ At the disc‐vertebra interface, we observed that local TMD and BV/TV gradients weakly associated with interface strength, although the magnitude of TMD and BV/TV at the endplates was a more significant predictor. Future studies should investigate functional grading across the entire EPJ (annulus‐CEP‐bone). For example, nano‐indentation could be used to investigate stiffness of each involved tissue, and whether gradients in stiffness affect attachment strength.

In summary, we have measured failure strength, characterized failure mechanisms, assessed nanoscale structural connectivity, and uncovered structure‐function relationships at the human disc‐vertebra interface. Our results highlight the vulnerability of the CEP to tensile failure because of poor structural integration with the subchondral bone. We have also shown that disc degeneration grade, vertebral endplate bone density, and CEP thickness are all important predictors of disc‐vertebra failure strength. Our observations reinforce the need for diagnostic tests to characterize the integrity of this region, and activity modification to reduce the tensile stresses at this interface in at‐risk individuals. Further, these data motivate the development of treatments that target this region.

## AUTHORS’ CONTRIBUTIONS

B.B.J. designed and performed the experiments, analyzed all data, interpreted results, and wrote the manuscript. E.C.L. assisted in performing and interpreting histology, and A.L. performed μct scanning and image reconstruction. A.J.F and J.C.L. prepared the supporting grant, provided advice for experimental design and analysis, and critically revised the manuscript. All authors have read and approved the final submitted manuscript.
